# Risk of cancer following primary care presentation with fatigue: a population-based cohort study of a quarter of a million patients

**DOI:** 10.1038/s41416-022-01733-6

**Published:** 2022-02-18

**Authors:** Becky White, Meena Rafiq, Arturo Gonzalez-Izquierdo, Willie Hamilton, Sarah Price, Georgios Lyratzopoulos

**Affiliations:** 1 Epidemiology of Cancer Healthcare and Outcomes (ECHO) Research Group, Department of Behavioural Science and Health, University College London, London, UK; 2 Institute of Health Informatics, Faculty of Population Health Sciences, University College London, London, UK; 3 University of Exeter Medical School, St Luke’s Campus, Magdalen Road, Exeter, EX1 2LU UK

**Keywords:** Risk factors, Diagnosis, Fatigue, Cancer epidemiology

## Abstract

**Background:**

The management of adults presenting with fatigue presents a diagnostic challenge, particularly regarding possible underlying cancer.

**Methods:**

Using electronic health records, we examined cancer risk in patients presenting to primary care with new-onset fatigue in England during 2007–2013, compared to general population estimates. We examined variation by age, sex, deprivation, and time following presentation.

**Findings:**

Of 250,606 patients presenting with fatigue, 12-month cancer risk exceeded 3% in men aged 65 and over and women aged 80 and over, and 6% in men aged 80 and over. Nearly half (47%) of cancers were diagnosed within 3 months from first fatigue presentation. Site-specific cancer risk was higher than the general population for most cancers studied, with greatest relative increases for leukaemia, pancreatic and brain cancers.

**Conclusions:**

In older patients, new-onset fatigue is associated with cancer risk exceeding current thresholds for urgent specialist referral. Future research should consider how risk is modified by the presence or absence of other signs and symptoms. Excess cancer risk wanes rapidly after 3 months, which could inform the duration of a ‘safety-netting’ period. Fatigue presentation is not strongly predictive of any single cancer, although certain cancers are over-represented; this knowledge can help prioritise diagnostic strategies.

## Background

Many cancer patients are diagnosed after presenting to a general practitioner with non-site specific symptoms of relatively low specificity [1], for which there are limited referral or investigation guidelines [2]. Cancer patients least likely to be diagnosed following fast-track referral are those with cancers typically characterised by such non-site specific presenting symptoms (e.g. fatigue, weight loss etc.), which have low positive predictive value (PPV) for any single cancer [3]. Consequently, these patients often experience prolonged intervals before diagnosis [2].

Fatigue is a relatively common presenting symptom in primary care, being the principal complaint in an estimated 5–7% of consultations [4–7]. It is even more common in the general population, with 15–40% of people reporting experiencing fatigue in the last two or four weeks [8, 9]. Fatigue is known to be a presenting feature of several cancers [10–13]. Diagnostic guidelines by the National Institute for Health and Care Excellence (NICE) regarding fatigue recommend urgent 2-week-wait referral only for specific presentations where available evidence shows the positive predictive value (PPV) for specific cancer diagnosis (usually within 12 months) exceeds 3% [12–14]. However, the range of cancer sites associated with fatigue and their relative specific risk is not adequately described in current literature, which is dominated by studies focusing on individual cancer sites. Nonetheless, the limited available evidence suggests that the predictive value of fatigue as a single presenting symptom for colorectal, lung, or urological cancers and for leukaemia is likely to be low [12, 15–17]. As a relatively common symptom, fatigue can also signal a range of other conditions, including but not limited to: self-limiting illnesses (e.g. short-term post-viral fatigue); chronic fatigue syndrome; depression; a range of other diseases (e.g. hypothyroidism, vitamin deficiency, iron deficiency, coeliac disease etc.); and more rarely, autoimmune disease such as lupus or chronic infection such as hepatitis C [5, 18–21].

Given the low PPV of fatigue for cancer, and the range of possible other causes, primary and secondary care clinicians must assess which patients presenting with fatigue are more likely to have cancer, thereby requiring specialist referral. Consequently, investigating the predictive value of fatigue for any cancer and specific types of cancer, for different age and sex groups, is important to help determine appropriate diagnostic strategies to diagnose or rule out specific cancers efficiently. Although patients who seek medical help for fatigue are not representative of the broader population of individuals with fatigue in the community [8, 9, 22], understanding their cancer risk when they first present to primary care is important to support general practitioners’ decisions about their management. It is also unclear how long patients who present with new-onset fatigue remain at greater risk of being diagnosed with cancer after initial presentation, and therefore how long healthcare professionals and patients should be alert to changing symptoms and other diagnostic clues (i.e. the ‘safety-netting’ period) [23].

Therefore, this study aimed to establish the risk of present but as-yet-undetected cancer (overall and by specific cancer site) among patients who present with ‘new onset’ fatigue to a general practitioner, and related changes over time in such risks in the months after initial presentation. It also aimed to contextualise the excess risk in these patients through comparisons with cancer risk in the general population for persons of the same sex and age band.

## Methods

### Study design and data source

We conducted a cohort study of patients with a record of fatigue presentation in primary care in England between 2007 and 2013, using electronic health records (EHRs) from the Clinical Practice Research Datalink (CPRD) GOLD (March 2019 database build). CPRD stores data about patients presenting to primary care for approximately 6.9% (*N* = 4.4 million) of the UK population in 2013 [24]. Data include patients’ recorded symptoms and socio-demographic information (age, sex). The Index of Multiple Deprivation (IMD) quintile of the patient’s neighbourhood of residence was identified, through linkage via the patient’s postcode. Cancers diagnosed from 2006 to 2015 in this cohort of patients were identified through linkage with national cancer registration data held by the National Cancer Registration & Analysis Service (NCRAS), using an eight-step deterministic linkage algorithm including NHS number, sex, date of birth, and postcode.

### Cohort identification

In step 1, patients were included in the study if they had a code for fatigue recorded during a consultation in CPRD within the overall study period (2007–2013). In step 2, patients were included if at least one of their fatigue records was ‘eligible’, i.e. the date occurred *after* all of the following events: the date the patient’s practice was up to standard regarding research quality, the patient was registered to the practice for at least a year, and the patient was 30 years old. The date also had to occur before all of the following events (if relevant): the date the practice last submitted data to CPRD, the patient left the practice, the patient was aged 100 years or over, or the patient’s death.

We aimed to ensure the study population broadly represented patients attending primary care with new-onset fatigue, to minimise the likelihood that it was attributable to a previously diagnosed condition or disease (including cancer). Therefore, in step 3, we excluded a small group of patients who had an ‘ineligible’ record of fatigue in the year before their first eligible fatigue record (as a patient could have had a prior record of fatigue before the date they entered the study as defined in step 2 (e.g. before the patient was 30 years old)). This meant that patients did not enter the study midway through a series of consultations for fatigue. However, if such patients had another eligible fatigue record more than a year later, the later record was selected and the patient was included.

**Fig. 1:**
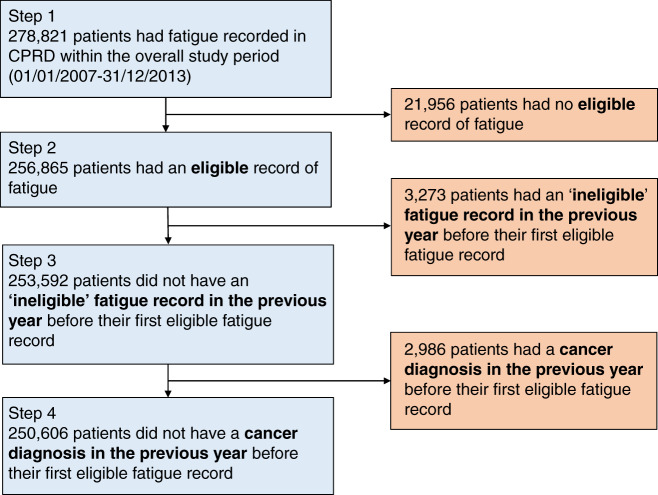
Study inclusions and exclusions. Numbers of patients included (left) and excluded (right) at each step are shown.

To minimise the likelihood that fatigue was attributable to a previous cancer diagnosis, in step 4, we also excluded patients if there was a cancer diagnosis recorded in NCRAS in the year before or on the same day as their first eligible fatigue record. In these cases, the patient was still included if they had another eligible fatigue record more than a year after the first eligible fatigue record. We conducted a sensitivity analysis where we extended the look-back period in steps 3 and 4 to 2 years, and also where these two exclusions were not conducted. In Results, Fig. 1 illustrates steps 1–4.

According to NICE Guidelines [25], there is no universal definition of fatigue. Therefore, WH and SP developed medical code lists used to identify fatigue, using methods detailed by Watson et al. [26] (Supplementary Appendix 1). Although the study was concerned with new onset fatigue, we included codes for chronic fatigue syndrome (CFS) and post viral fatigue syndrome (PVFS), because some patients with fatigue who may have cancer could initially be misdiagnosed with CFS or PVFS. This is analogous to previous research, which has highlighted possible misdiagnoses of Irritable Bowel Syndrome (IBS) in some patients subsequently diagnosed with colorectal cancer [27, 28]. Nonetheless, we conducted a sensitivity analysis to ascertain whether excluding CFS and PVFS impacted cancer risk estimates. We did not consider other pre-existing conditions (e.g. anaemia) that could have explained the presence of fatigue.

### Follow up and outcomes

Follow up began with the patient’s first eligible record of fatigue during the study period (termed the ‘index’ record). Follow up ended either at 1 year following the index record, or at first cancer diagnosis, whichever was earlier. As NCRAS data was used to define the outcome, patients could remain in the study even after they had left their GP practice or their practice had exited CPRD. After follow up ended, patients could not re-enter the study with a subsequent fatigue record (i.e. patients were included in the study once).

The main outcome was diagnosis of cancer recorded in cancer registry (NCRAS) data within 12 months after first (index) fatigue record. One year was chosen to enable comparison with most primary research underpinning diagnostic guidelines regarding fatigue (NICE) [12–14]. We conducted a supplementary analysis following patients up to 2 years, which confirmed that 1 year was long enough to capture relevant cancer cases (Supplementary Appendix 2). Cancers included any malignant neoplasms, excluding non-melanoma skin cancer (ICD-10 codes C00-C99 excl. C45). Benign brain tumours were not included. Cancer site definitions were adapted from previously published ICD-10 codes [29]. Rarer cancers were combined into anatomically related groups or, where this was not possible, grouped into ‘other cancers’ (Supplementary Appendix 3).

### Statistical analysis

The age, sex, and deprivation quintile of patients with fatigue were compared to the general population in England (Table 1, Supplementary Appendix 5). We calculated the risk of cancer, for all cancers combined and stratified by cancer site. Analyses were stratified by sex and 5-year age band, as there is substantial variability in cancer incidence by sex and age [30]. We aimed to estimate values to a level of precision where 95% confidence intervals were no wider than 0.5 percentage points either side of the cancer risk estimates. Assuming proportions of 3%, we calculated that sample sizes of at least 3,700 patients were needed in each age-sex strata.

**Table 1:** Gender and age characteristics of patients presenting to primary care with fatigue compared to general population estimates, by subsequent cancer diagnosis within a year after first presentation.

	Patients with fatigue				England population			
Age group^a^	No cancer		Cancer^b^		No cancer		Cancer^c^	
	*n*	%	*n*	%	*n*	%	*n*	%
*Men*								
30–34	4,737	5.98	<5^d^	–	1,764,208	11.13	1,074	0.79
35–39	6,194	7.83	12	0.60	1,754,358	11.07	1,299	0.95
40–44	7,600	9.60	19	0.96	1,919,735	12.11	2,183	1.60
45–49	8,147	10.29	30	1.51	1,922,296	12.13	3,804	2.78
50–54	7,960	10.06	80	4.03	1,692,822	10.68	6,166	4.51
55–59	7,864	9.93	117	5.89	1,474,698	9.30	10,086	7.38
60–64	8,553	10.81	208	10.47	1,534,388	9.68	17,610	12.88
65–69	6,742	8.52	254	12.78	1,221,416	7.71	21,197	15.51
70–74	6,270	7.92	298	15.00	941,209	5.94	22,369	16.36
75–79	6,040	7.63	362	18.22	739,908	4.67	21,219	15.52
80–84	4,744	5.99	328	16.51	507,744	3.20	16,448	12.03
85+	4,305	5.44	275	13.84	376,656	2.38	13,253	9.69
Mean, median age	58, 58		73, 74		54, 52		70, 71	
Total men	79,156		1987		15,849,438		136,708	
*Women*								
30–34	16,215	9.69	22	1.05	1,762,200	11.13	1,668	0.79
35–39	18,706	11.18	32	1.52	1,764,022	11.07	2,693	0.95
40–44	20,142	12.03	59	2.81	1,954,742	12.11	4,823	1.60
45–49	19,866	11.87	99	4.71	1,958,271	12.13	7,705	2.78
50–54	16,628	9.94	119	5.67	1,713,902	10.68	9,689	4.51
55–59	13,851	8.28	137	6.52	1,508,030	9.30	10,687	7.38
60–64	12,591	7.52	219	10.43	1,594,525	9.68	15,768	12.88
65–69	10,523	6.29	231	11.00	1,298,592	7.71	16,415	15.51
70–74	10,146	6.06	238	11.33	1,055,292	5.94	15,681	16.36
75–79	10,432	6.23	316	15.05	901,399	4.67	15,805	15.52
80–84	8,654	5.17	307	14.62	726,834	3.20	14,821	12.03
85+	9,609	5.74	321	15.29	786,631	2.38	16,778	9.69
Mean, median age	55, 52		70, 72		55, 53		68, 69	
Total women	167,363		2,100		17,024,440		132,533	

^a^Age at first presentation. Mean and median ages for available population estimates were estimated from aggregated 5-year age bands.

^b^Cancer diagnoses between 2007 and 2014, 12 months after first presentation with fatigue to primary care in 2007–2013, while aged 30–99 years.

^c^Estimated 12-month population incidence, based on annual number of cancer diagnoses and mid-year population estimates for England, 2011. Available population estimates include patients aged > 99 years. This was estimated to account for < 0.9% of people aged 85+ in this analysis, thus would have a negligible impact on cancer incidence estimates for this age group.

^d^Cell counts under 5 are suppressed to reduce statistical disclosure risk.

**Fig. 2:**
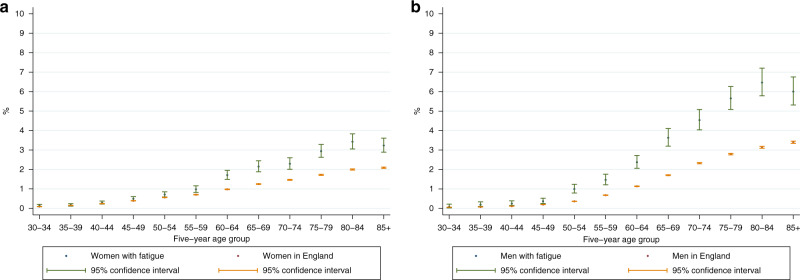
Cancer risk (%) within a year among patients with fatigue, compared to general population in England. **a** Men. **b** Women.

For Table 2 and Fig. 2, we calculated absolute and relative differences in cancer risk between patients with fatigue and the general population (derived using incident cancer registration statistics for England in 2011 [31] and corresponding mid-year population estimates) [32], for each age-sex stratum. For calculations using the general population estimates, we assumed that no person was diagnosed with more than one cancer during a year. We conducted a separate supplementary analysis of cancer risk by deprivation quintile, as there were no directly comparable general population cancer risk estimates that would have allowed us to also adjust for age and sex.

**Table 2:** Number and proportion of patients diagnosed with cancer within a year after presenting to primary care with fatigue compared to general population estimates, by gender and age band.

	Patients with fatigue			England population					
	Cancer^a^		Total	Cancer^b^		Total			
	*n*	% [lci,uci]	*N*	*n*	% [lci,uci]	*N*	Absolute difference (%)	Risk ratio [lci,uci]	*P*-value
*Men*									
30–34	<5^c^	–	4,741	1,074	0.06 [0.06,0.06]	1,765,282	–	–	–
35–39	12	0.19 [0.1,0.34]	6,206	1,299	0.07 [0.07,0.08]	1,755,657	0.12	2.61 [1.35,4.58]	0.001
40–44	19	0.25 [0.15,0.39]	7,619	2,183	0.11 [0.11,0.12]	1,921,918	0.14	2.2 [1.32,3.44]	0.001
45–49	30	0.37 [0.25,0.52]	8,177	3,804	0.2 [0.19,0.2]	1,926,100	0.17	1.86 [1.25,2.66]	0.001
50–54	80	1 [0.79,1.24]	8,040	6,166	0.36 [0.35,0.37]	1,698,988	0.63	2.74 [2.17,3.42]	<0.001
55–59	117	1.47 [1.21,1.76]	7,981	10,086	0.68 [0.67,0.69]	1,484,784	0.79	2.16 [1.78,2.59]	<0.001
60–64	208	2.37 [2.06,2.72]	8,761	17,610	1.13 [1.12,1.15]	1,551,998	1.24	2.09 [1.82,2.4]	<0.001
65–69	254	3.63 [3.2,4.11]	6,996	21,197	1.71 [1.68,1.73]	1,242,613	1.92	2.13 [1.87,2.41]	<0.001
70–74	298	4.54 [4.04,5.08]	6,568	22,369	2.32 [2.29,2.35]	963,578	2.22	1.95 [1.74,2.19]	<0.001
75–79	362	5.65 [5.09,6.27]	6,402	21,219	2.79 [2.75,2.83]	761,127	2.87	2.03 [1.82,2.25]	<0.001
80–84	328	6.47 [5.79,7.21]	5,072	16,448	3.14 [3.09,3.19]	524,192	3.33	2.06 [1.84,2.3]	<0.001
85+	275	6 [5.32,6.76]	4,580	13,253	3.4 [3.34,3.46]	389,909	2.61	1.77 [1.56,1.99]	<0.001
*Women*									
30–34	22	0.14 [0.08,0.21]	16,237	1,668	0.09 [0.09,0.1]	1,763,868	0.04	1.43 [0.9,2.18]	0.092
35–39	32	0.17 [0.12,0.24]	18,738	2,693	0.15 [0.15,0.16]	1,766,715	0.02	1.12 [0.76,1.59]	0.522
40–44	59	0.29 [0.22,0.38]	20,201	4,823	0.25 [0.24,0.25]	1,959,565	0.05	1.19 [0.9,1.53]	0.190
45–49	99	0.5 [0.4,0.6]	19,965	7,705	0.39 [0.38,0.4]	1,965,976	0.10	1.27 [1.03,1.54]	0.020
50–54	119	0.71 [0.59,0.85]	16,747	9,689	0.56 [0.55,0.57]	1,723,591	0.15	1.26 [1.05,1.51]	0.011
55–59	137	0.98 [0.82,1.16]	13,988	10,687	0.7 [0.69,0.72]	1,518,717	0.28	1.39 [1.17,1.65]	<0.001
60–64	219	1.71 [1.49,1.95]	12,810	15,768	0.98 [0.96,0.99]	1,610,293	0.73	1.75 [1.52,2]	<0.001
65–69	231	2.15 [1.88,2.44]	10,754	16,415	1.25 [1.23,1.27]	1,315,007	0.90	1.72 [1.5,1.96]	<0.001
70–74	238	2.29 [2.01,2.6]	10,384	15,681	1.46 [1.44,1.49]	1,070,973	0.83	1.57 [1.37,1.78]	<0.001
75–79	316	2.94 [2.62,3.28]	10,748	15,805	1.72 [1.7,1.75]	917,204	1.22	1.71 [1.52,1.91]	<0.001
80–84	307	3.43 [3.05,3.83]	8,961	14,821	2 [1.97,2.03]	741,655	1.43	1.71 [1.53,1.92]	<0.001
85+	321	3.23 [2.89,3.61]	9,930	16,778	2.09 [2.06,2.12]	803,409	1.14	1.55 [1.38,1.73]	<0.001

^a^Cancer diagnoses between 2007 and 2014, 12 months after first presentation with fatigue to primary care in 2007–2013, while aged 30–99 years.

^b^Estimated 12-month population incidence, based on annual number of cancer diagnoses and mid-year population estimates for England, 2011. Available population estimates include patients aged >99 years. This was estimated to account for <0.9% of people aged 85+ in this analysis, thus would have a negligible impact on cancer incidence estimates for this age group. ^c^Cell counts under 5 are suppressed to reduce statistical disclosure risk.

For secondary analyses, we also used general population estimates to derive expected cancer risk for all persons with fatigue, as described in prior literature [33–35]. We directly standardised general population estimates by multiplying the total number of patients in each sex and 5-year age band in the fatigue cohort by the corresponding annual cancer incidence in the general population, thereby obtaining the expected age- and sex-specific number of incident cancers in the fatigue cohort. These were summed to calculate expected cancers for men, women, and both sexes combined.

For Table 3, we anticipated that risk estimates would have adequate precision for the comparison between observed and expected risk for certain cancer sites, though estimates for particularly rare cancer sites (under 30 cases) were not shown.

**Table 3:** First cancer site diagnosed within a year, as a proportion of patients presenting to primary care with fatigue, observed compared to expected.

	Observed^a^		Expected^b^				
	*n*	% [lci,uci]	n	% [lci,uci]	Absolute difference (%)	Risk ratio [lci,uci]	*P*-value
*Men*							
Chi2 (*P*-value) comparing observed and expected distribution of cancer sites: <0.001							
All cancers	1,987	2.45 [2.34,2.56]	980	1.21 [1.2,1.21]	1.24	2.03 [1.94,2.12]	<0.001
Prostate	406	0.5 [0.45,0.55]	255	0.31 [0.31,0.32]	0.19	1.59 [1.44,1.75]	<0.001
Lung and mesothelioma	384	0.47 [0.43,0.52]	159	0.2 [0.19,0.2]	0.28	2.41 [2.18,2.67]	<0.001
Colorectal	251	0.31 [0.27,0.35]	138	0.17 [0.17,0.17]	0.14	1.82 [1.61,2.06]	<0.001
Upper gastro-intestinal	125	0.15 [0.13,0.18]	62	0.08 [0.07,0.08]	0.08	2.03 [1.7,2.42]	<0.001
Lymphoma	121	0.15 [0.12,0.18]	42	0.05 [0.05,0.05]	0.10	2.89 [2.41,3.45]	<0.001
Leukaemia	98	0.12 [0.1,0.15]	28	0.03 [0.03,0.04]	0.09	3.49 [2.86,4.27]	<0.001
Unknown primary	83	0.1 [0.08,0.13]	29	0.04 [0.03,0.04]	0.07	2.87 [2.31,3.57]	<0.001
Pancreas	81	0.1 [0.08,0.12]	27	0.03 [0.03,0.03]	0.07	3.03 [2.43,3.78]	<0.001
Kidney	71	0.09 [0.07,0.11]	29	0.04 [0.03,0.04]	0.05	2.43 [1.92,3.07]	<0.001
Other malignant neoplasms	65	0.08 [0.06,0.1]	28	0.04 [0.03,0.04]	0.04	2.28 [1.79,2.92]	<0.001
Bladder	56	0.07 [0.05,0.09]	50	0.06 [0.06,0.06]	0.01	1.11 [0.85,1.44]	0.438
Brain and other CNS	55	0.07 [0.05,0.09]	13	0.02 [0.02,0.02]	0.05	4.25 [3.25,5.55]	<0.001
Melanoma	43	0.05 [0.04,0.07]	34	0.04 [0.04,0.04]	0.01	1.25 [0.93,1.69]	0.146
Multiple myeloma	41	0.05 [0.04,0.07]	17	0.02 [0.02,0.02]	0.03	2.48 [1.82,3.38]	<0.001
Liver	40	0.05 [0.04,0.07]	16	0.02 [0.02,0.02]	0.03	2.5 [1.83,3.42]	<0.001
Head and neck	39	0.05 [0.03,0.07]	35	0.04 [0.04,0.04]	0.00	1.11 [0.81,1.52]	0.523
Sarcoma	<30	–	–	–	–	–	–
Thyroid	<30	–	–	–	–	–	–
Testis	<30	–	–	–	–	–	–
Breast	<30	–	–	–	–	–	–
Total men	81,143		81,143				
*Women*							
Chi2 (*P*-value) comparing observed and expected distribution of cancer sites: <0.001							
All cancers	2,100	1.24 [1.19,1.29]	1,348	0.8 [0.79,0.8]	0.44	1.56 [1.49,1.63]	<0.001
Breast	426	0.25 [0.23,0.28]	409	0.24 [0.24,0.24]	0.01	1.04 [0.95,1.15]	0.408
Lung and mesothelioma	328	0.19 [0.17,0.22]	165	0.1 [0.1,0.1]	0.10	1.99 [1.78,2.22]	<0.001
Colorectal	301	0.18 [0.16,0.2]	157	0.09 [0.09,0.09]	0.09	1.92 [1.71,2.15]	<0.001
Lymphoma	112	0.07 [0.05,0.08]	53	0.03 [0.03,0.03]	0.03	2.1 [1.75,2.54]	<0.001
Pancreas	105	0.06 [0.05,0.08]	39	0.02 [0.02,0.02]	0.04	2.71 [2.23,3.29]	<0.001
Unknown primary	101	0.06 [0.05,0.07]	47	0.03 [0.03,0.03]	0.03	2.13 [1.75,2.6]	<0.001
Ovary	95	0.06 [0.05,0.07]	57	0.03 [0.03,0.03]	0.02	1.65 [1.35,2.02]	<0.001
Uterus	77	0.05 [0.04,0.06]	69	0.04 [0.04,0.04]	0.00	1.12 [0.9,1.41]	0.313
Brain and other CNS	66	0.04 [0.03,0.05]	17	0.01 [0.01,0.01]	0.03	3.97 [3.11,5.08]	<0.001
Leukaemia	65	0.04 [0.03,0.05]	29	0.02 [0.02,0.02]	0.02	2.23 [1.75,2.86]	<0.001
Melanoma	65	0.04 [0.03,0.05]	54	0.03 [0.03,0.03]	0.01	1.21 [0.94,1.54]	0.133
Upper gastro-intestinal	64	0.04 [0.03,0.05]	45	0.03 [0.03,0.03]	0.01	1.43 [1.12,1.83]	0.004
Kidney	56	0.03 [0.02,0.04]	27	0.02 [0.02,0.02]	0.02	2.06 [1.58,2.68]	<0.001
Other malignant neoplasms	56	0.03 [0.02,0.04]	39	0.02 [0.02,0.02]	0.01	1.43 [1.1,1.86]	0.008
Bladder	31	0.02 [0.01,0.03]	27	0.02 [0.02,0.02]	0.00	1.15 [0.81,1.64]	0.447
Liver	30	0.02 [0.01,0.03]	14	0.01 [0.01,0.01]	0.01	2.21 [1.54,3.18]	<0.001
Multiple myeloma	30	0.02 [0.01,0.03]	18	0.01 [0.01,0.01]	0.01	1.62 [1.13,2.33]	0.008
Cervix	<30	–	–	–	–	–	–
Thyroid	<30	–	–	–	–	–	–
Sarcoma	<30	–	–	–	–	–	–
Head and neck	<30	–	–	–	–	–	–
Vulva	<30	–	–	–	–	–	–
Total women	169,463		169,463				

^a^Cancer diagnoses between 2007–2014, 12 months after first presentation with fatigue to primary care in 2007–2013.

^b^Expected cases for the age distribution of men and women with fatigue, based on 5-year age band and sex-specific estimated monthly population incidence, using annual number of cancer diagnoses and mid-year population estimates for England, 2011. Results not shown for cancers with fewer than 30 observed cases.

**Fig. 3:**
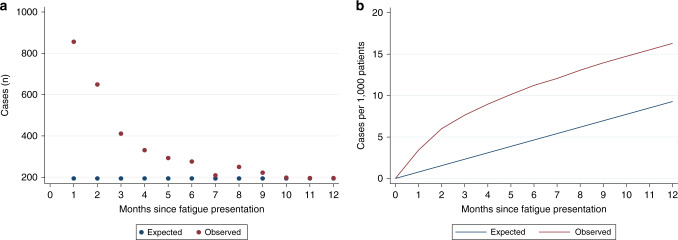
Number of cancer cases by month after first presentation with fatigue, compared to general population in England. **a** Monthly cancer cases (*n*). **b** Monthly cumulative rate of cases per 1000 patients.

To better describe variability in excess cancer risk after the initial record of fatigue, in Fig. 3 and Supplementary Appendix 2, we compared the observed and expected number of cancer cases by month of follow-up. To derive expected monthly cases, annual cancer incidence in the general population was divided by 12 and then age- and sex-standardised to derive expected monthly cases. We subtracted the expected from the observed monthly cases, to calculate excess cases each month.

Data management was conducted in MySQL Workbench version 6.1, with all statistical analysis conducted in Stata version 16. Age and sex standardisation was performed using the user-written distrate command for Stata [36], with 95% confidence intervals calculated using the Dobson et al. method for rare outcomes [37]. Pearson’s chi-square tests (which were robust to assumptions about data distribution and degree of homoscedasticity) were used to assess statistical significance of differences in cancer incidence between the fatigue cohort and the general population. *P* values < 0.05 were considered significant. We used Strengthening the Reporting of Observational studies in Epidemiology (STROBE) guidelines for cohort studies [38] to report this study (Supplementary Appendix 4).

## Findings

### Cohort description

Of the 278,821 individuals who had a record of fatigue in primary care between 2007 and 2013, 250,606 (90%) had at least one ‘eligible’ record within the patient’s inclusion period, without either a cancer diagnosis or an ‘ineligible’ fatigue record in the previous year (Fig. 1). These were included in the study cohort. There was a preponderance of women in the cohort (68%), compared to 52% in the general population [32]. The study cohort was also slightly older than the general population. For example, among patients with fatigue subsequently diagnosed with cancer, 49% of men and 45% of women were aged 75 years and over, compared to 37% and 37% in the general population, respectively (Table 1). The study cohort was also slightly less deprived, with 23% in the least deprived quintile compared to 18% of people aged 30 years and over in the general population (Supplementary Appendix 5). Regarding specific subcodes, 0.81% (*n* = 2,033) of the study cohort had a first fatigue record for either Chronic Fatigue Syndrome (CFS) or post viral fatigue syndrome (PVFS) (Supplementary Appendix 6).

### Risk of cancer

For men, the risk of any cancer diagnosis within a year after the first fatigue record ranged from below 1% in each 5-year age band from those aged 30–49, to 3–6% in age bands between 65–79 years, and over 6% for those aged 80 years and over (Fig. 2a, Table 2). Cancer risk was higher in men with fatigue than men in the general population in every age band from 35 years and over (*p* < 0.01 for all), and was typically at least twice as high, with no clear trend by age.

The risk of cancer in women with fatigue ranged from below 1% in those aged 30–59 years, to over 3% in those aged 80 years and over (Fig. 2b, Table 2). Cancer risk was higher in women with fatigue than women in the general population in every age band from 45 years and over (*p* < 0.05 for all), rising to between 55 and 75% higher among those aged 60 years and over.

Comparing patterns in men and women who presented with fatigue, the relative increases in cancer risk compared to the general population appeared higher in men than women. In each age band, risk ratios comparing men with fatigue to men in the general population were higher than risk ratios comparing women with fatigue to women in the general population; however, confidence intervals around these risk ratios generally indicated they were not statistically different (Table 2). In supplementary analysis, cancer risk among fatigue presenters was similar across deprivation quintiles (Supplementary Appendix 5).

### Frequency of specific cancer sites

For men, site-specific cancer risk was higher than expected for 13 of the 16 cancer sites studied (all *p* values < 0.001). Although their absolute associated risk was low (≤0.12%), in relative terms the observed risk of diagnosis of leukaemia, pancreatic, and brain cancers was 3- to 4-fold greater than expected (*p* < 0.001). The overall case mix of cancer sites was different to expected (*p* < 0.001), although the three most common cancers in men in the general population (prostate, lung and colorectal) still accounted for the majority (52% (*n* = 1041)) of observed cases in our sample (Table 3).

For women, site-specific cancer risk was higher than expected for 13 of the 17 cancer sites studied (all *p* values < 0.02). Although their absolute associated risk was low (≤0.06%), in relative terms, the observed risk of diagnosis of leukaemia, pancreatic, and brain cancers was 2- to 4-fold greater than expected (*p* < 0.001). The overall case mix of cancers was different to expected (*p* < 0.001), although the three most common cancers in women (breast, lung and colorectal cancers) in the general population still accounted for half (50%, *n* = 1055) of observed cases in our sample (Table 3).

### Distribution of incident cases by month following fatigue presentation

Of 4087 patients diagnosed with cancer within a year after their first fatigue record, 47% were diagnosed in the first 3 months. The number of excess cancer cases among patients with fatigue was greatest in the first month after the index fatigue record, when 856 new cases were observed, compared to 194 expected (*p* < 0.001). There followed a steep decrease in the number of cases until month nine, after which the observed count of monthly cancer cases was similar to expected (month 10, *p* = 0.77) (Fig. 3a, see also Supplementary Appendix 2 for follow up to 24 months). This was mirrored by a steep initial increase in the cumulative rate of excess cases. By month nine, in patients with fatigue, there were 14 cancer cases per 1000 patients, compared to an expected 7 per 1000. By month 12, there were 16 observed cases per 1000 patients, compared to 9 expected cases per 1000 patients (Fig. 3b, Supplementary Appendix 2).

### Sensitivity analyses

Sensitivity analysis showed that cancer risk was similar regardless of whether patients with either a cancer diagnosis or another fatigue record in the previous year were included, or whether the look-back period for exclusion was extended to 2 years (Supplementary Appendix 7). In further sensitivity analysis, excluding patients with CFS or PVFS codes from analysis produced similar results overall compared to including them, although cancer risk was lower in patients with CFS or PVF than in other patients with fatigue (Supplementary Appendix 6).

## Discussion

### Key findings

The risk of cancer diagnosis within a year following a primary care consultation with fatigue exceeded 3% among men aged 65 and over and women aged 80 and over, and 6% in men aged 80 and over. Cancer risk was at least two-fold greater than that of the general population in men across all age groups, and from 1.5- to 1.7-fold greater than that of the general population in women aged 60 years and over. Although the risk was greater than expected for most cancers, certain cancers, such as leukaemia, pancreatic cancer, and brain cancers, were over-represented among patients with fatigue. Cancer risk was greatest in the 3 months following the initial presentation, and was three times higher than expected in the first month, but returned to the background rate by nine months.

### Strengths and limitations

This study used high quality primary care records from CPRD, which is broadly representative of the UK population regarding age, sex, and ethnicity, although may not be representative of all GP practices based on geography and size [24]. Full coverage of cancer diagnoses for the study cohort was possible, via linkage to ‘gold standard’ population-level cancer registration data (NCRAS) [39]. The large cohort produced precise estimates of cancer risk by sex and age band, although estimates for rarer cancer sites (e.g. head and neck cancers) may have lacked precision for comparisons between observed and expected risk.

Some instances of a patient’s presentation with fatigue may not be recorded by the GP, due to variation in coding practices. GPs are more likely to record alarm symptoms as coded entries rather than free text (which is not generally available to researchers) when there is a suspicion for cancer [40]. If coded recording of alarm symptoms is more common in patients who are subsequently diagnosed with cancer than for those who are not, this would artificially inflate cancer risk estimates for alarm symptoms. While patterns of coded recording for fatigue are not known, under-recording of abdominal pain (which, like fatigue, is a non-specific symptom) was similar in people with and without cancer, which meant that cancer risk estimates were not inflated [40].

Our study focuses on patients with fatigue who have sought medical help, and is not generalisable to people experiencing fatigue in the community [9]. The comparisons we have made to the general population should be interpreted as contextualising cancer risk among patients presenting to GPs with fatigue, above what would usually be expected for their age and sex. Increased cancer risk may in part reflect differences in the characteristics of patients who consult primary care [9].

Cancer risk in the general population could also be slightly overestimated, as we assumed that one cancer case in the published estimates equalled one person (i.e. that there were no persons with multiple primary diagnoses). Population incidence estimates also include cancers diagnosed in patients who have presented with fatigue, again making our comparisons of observed versus expected incidence conservative.

To produce risk estimates relevant to primary care clinicians, we aimed to ensure the study population broadly represented patients attending primary care with new-onset fatigue, minimising the likelihood that it was attributable to a previously diagnosed condition or disease (including cancer) or its treatment. Therefore, we excluded patients if all of their potential index fatigue records occurred within a year following another fatigue record or a cancer diagnosis. Nonetheless, a sensitivity analysis showed that results were similar whether including or excluding these patients, and whether extending the look back period from 1 year before index record to 2 years.

We did not investigate fatigue in combination with other potential symptoms that could have been reported in the same or an earlier consultation, or related tests or investigations. In common with studies using electronic health records, it is not possible to infer whether the patient’s concern about fatigue was the primary reason for the encounter. It is, therefore, possible that some diagnoses were the result of investigations triggered by another potential cancer sign or symptom in the same consultation or an earlier consultation. In principle, this could partly contribute to the short time interval between first fatigue presentation and cancer diagnosis in a number of cases. Finally, the date of cancer diagnosis is defined by NCRAS according to hierarchical rules recommended by the European Network of Cancer Registries. In some cases, this is the date of pathological verification, and may be occurring later than the date the patient received the clinical diagnosis of cancer [41].

### Comparison with literature

Available evidence underpinning current NICE guidelines has so far only examined the positive predictive value (PPV) of fatigue for diagnosis of a small number of specific cancer sites [12–14]. Our study substantially enhances previous evidence regarding the risk of present but as-yet-undetected cancer among patients presenting to primary care with fatigue, as it is the first to examine risk of cancer overall, as well as several of the most common cancer sites diagnosed in these patients.

According to a systematic review, previous studies (generally using case-control designs) have found that fatigue was associated with specific cancers such as leukaemia, lung and kidney cancers [42]. However, a widely-used risk prediction tool (QCancer) reported that fatigue was not a significant independent predictor of cancer within 24-months, unlike other non-site specific symptoms, such as weight loss, appetite loss, and venous thrombo- embolism [43, 44]. Differences to our study could arise from various factors, including differences in the data source, length of follow-up, and adjustment for other presenting symptoms.

Few previous studies have sought to identify the most appropriate follow-up period to calculate subsequent cancer risk, though 12 or 24 month periods have been mostly used. One study demonstrated that patients presenting with weight loss (also a non-specific symptom) were at increased risk of a cancer diagnosis up to 3 months after initial presentation, with rapidly waning risk thereafter [45]. Our findings mirror this, as half of patients with underlying cancer were diagnosed in the first 3 months, although observed cancer risk remained substantially higher than expected for patients with fatigue for up to 9 months after the index fatigue record.

### Implications

Our study showed that overall 1-year cancer risk in patients presenting to primary care with new-onset fatigue was under 3% in men under 65 years, and women under 80. This suggests that, according to current guidelines, urgent two-week-wait referral for suspected cancer would not usually be necessary in these patients if simply considering the presence of fatigue. Notably, cancer risk in younger men (aged 50–64 years) and women (aged 60–75 years) presenting with fatigue was still relatively high compared to the general population. Patients deemed to be at low but not no risk of cancer should still be assessed in primary care, and where necessary investigated for suspected cancer via other urgent or non-urgent pathways, or actively monitored [23]. In future, such patients could also become eligible for 2-week-wait referral if risk thresholds were to be revised downwards (e.g. to 2%) [46].

Risk was greater than 3% in men aged 65 and over, and women aged 80 and over with new-onset fatigue, suggesting referral for suspected cancer may be appropriate in these groups. The benefits of ruling out serious physical disease such as cancer must be weighed against the risks of over investigation in older patients with non-specific symptoms, with appropriate communication of diagnostic uncertainty and guided by patient preferences [47].

In practice, patients with fatigue who also present with a site-specific ‘alarm’ symptom for cancer (e.g. breast lump, rectal bleeding, post-menopausal bleeding) are likely to be referred to an urgent two-week-wait pathway for suspected cancer under NICE Guidelines, and the diagnostic strategy is considerably clearer in these cases. Therefore, future research is needed to quantify how the increased cancer risk associated with new onset fatigue is modified by the presence or absence of co-occurring symptoms. In addition, for patients with fatigue who do not present with other, organ-specific, symptoms, future research could investigate which primary care tests (e.g. commonly used blood tests, chest X-ray, quantitative Faecal Immunochemical Test (qFIT)) could help to assess the risk of various common cancers. In England, such research could support the development of Rapid Diagnostic Centres (RDC), or similar initiatives regarding multidisciplinary diagnostic assessment one-stop services in other countries, which aim to expedite diagnosis in patients with non-specific symptoms such as fatigue [48].

Consistent with prior evidence, there were more women than men with fatigue identified in our CPRD population [4, 7, 43, 44, 49], which may reflect higher prevalence of conditions (other than cancer) associated with fatigue in women than men [49]. Alternatively, help-seeking behaviours may be different, with men being less likely to report potential cancer symptoms to primary care [9], resulting in an overrepresentation of men with severe fatigue indicating serious underlying physical disease such as cancer. Either of these mechanisms (or their combination) could explain why the observed risk in women was lower than that in men.

The findings relating to the relative frequency of cancer sites diagnosed (i.e. fatigue’s ‘cancer site signature’ [10]) can support the choice of suitable diagnostic test strategies (e.g. the ordering of tests) to most efficiently establish or rule out suspicion of the most likely cancers, when further assessment or referral is deemed appropriate. Our study reveals that the case mix of cancers in patients who presented with fatigue is different to that of incident cancer cases in the general population, although the most common cancers still accounted for large proportions of cases. No cancer site specific risk exceeded the NICE 3% 2-week-wait referral threshold, although leukaemia, pancreatic and brain cancers were particularly overrepresented among patients with fatigue, relative to their expected incidence. This could reflect cancer-specific pathophysiological mechanisms, for example, a high prevalence of anaemia leading to fatigue as a presenting symptom in patients with underlying leukaemia. However, we could not examine such biological pathways directly, and other explanatory mechanisms may be possible.

The findings suggest that should a clinician and patient decide to ‘actively monitor’ any potential cancer risk following the patient’s first presentation with fatigue (not considering other co-occurring symptoms), the length of this period should be up to 9 months, though most of this risk is concentrated in the first three. These represent periods when both healthcare professionals and patients should be vigilant of developing symptoms for cancer —though the risk of other (non-neoplastic disease) diagnoses should also be borne in mind. For the main analyses, we provided 1-year cancer risk estimates to facilitate comparison with existing NICE Guidelines. As the majority of excess cases occur soon after fatigue presentation, the difference between nine and 12-month risk estimates was small (0.2 percentage points, overall).

## Conclusions

Our study suggests that in men over 65 and women over 80, new onset fatigue is associated with cancer risk that exceeds current thresholds for urgent two-week wait referral. Future research should consider how risk is modified by the presence or absence of other signs and symptoms. Fatigue is associated with a broad range of cancer sites, but is not strongly predictive of any specific one, though certain cancers are more likely. Overall, excess cancer risk was concentrated in the first 3 months, though remaining comparatively greater than the general population up to 9 months following a new fatigue presentation, which could inform the duration of a surveillance period, when active monitoring is deemed appropriate.

## Supplementary information

Supplementary Appendices

## Data Availability

The data used in this study were accessed through the CPRD, and is subject to protocol approval by an Independent Scientific Advisory Committee, and therefore cannot be directly shared.

## Code availability

Code lists used to define fatigue are available in Supplementary Appendix 1.
