# Supplementary material for: Risk of cancer following primary care presentation with fatigue: a population-based cohort study of a quarter of a million patients
Source: Br J Cancer. 2022 Feb 18;126(11):1627–36. doi: 10.1038/s41416-022-01733-6 (PMC9130200; doi:10.1038/s41416-022-01733-6)
Supplement: Supplementary file 1 — Supplementary Appendices [file 41416_2022_1733_MOESM1_ESM.docx]

**Supplementary appendices**

**Appendix 1. Read codes used to define fatigue**

| CPRD Medcode | Readcode | Read term |
| --- | --- | --- |
| 5794 | 168..00 | Tiredness symptom |
| 5751 | 1683 | Tired all the time |
| 1147 | R007500 | [D]Tiredness |
| 15516 | 1683.11 | C/O - 'tired all the time' |
| 5583 | 168..12 | Lethargy - symptom |
| 1404 | 1682 | Fatigue |
| 6029 | 1B3..12 | Weakness symptoms |
| 7235 | E205.12 | Tired all the time |
| 6242 | 168..11 | Fatigue - symptom |
| 5658 | R007000 | [D]Malaise |
| 1371 | R007300 | [D]Lethargy |
| 7529 | F286.11 | CFS - Chronic fatigue syndrome |
| 1688 | R007100 | [D]Fatigue |
| 4546 | F286.00 | Chronic fatigue syndrome |
| 17736 | 1684 | Malaise/lethargy |
| 1900 | R2y3.00 | [D]Debility, unspecified |
| 9220 | 1688 | Exhaustion |
| 2855 | 1B32.00 | Weakness present |
| 5814 | R007z11 | [D]Lassitude |
| 9823 | 1684.11 | C/O - debility - malaise |
| 29292 | 168Z.00 | Tiredness symptom NOS |
| 6190 | F286.12 | Postviral fatigue syndrome |
| 9889 | R007211 | [D]General weakness |
| 1582 | E205.11 | Nervous exhaustion |
| 5049 | R007200 | [D]Asthenia NOS |
| 1042 | R007400 | [D]Postviral (asthenic) syndrome |
| 16479 | 2832.12 | O/E - weakness |
| 3361 | E205.00 | Neurasthenia - nervous debility |
| 9127 | F286.14 | Post-viral fatigue syndrome |
| 9656 | Eu46011 | [X]Fatigue syndrome |
| 23932 | R007z00 | [D]Malaise and fatigue NOS |
| 9435 | 2254 | O/E - apathetic |
| 12411 | R007411 | [D]Post viral debility |
| 27877 | F286.13 | PVFS - Postviral fatigue syn |
| 16561 | Eu46000 | [X]Neurasthenia |
| 44215 | R007.00 | [D]Malaise and fatigue |
| 24382 | R204.00 | [D]Senile exhaustion |
| 97284 | F286100 | Moderate chronic fatigue syndrome |
| 98512 | F286000 | Mild chronic fatigue syndrome |
| 98734 | F286200 | Severe chronic fatigue syndrome |

**Appendix 2. Number and cumulative proportion of patients with fatigue diagnosed with cancer, by month of first cancer diagnosis, observed compared to expected**

| Months | Observed^a^ | | | Expected^b^ | | | Excess cases | | Cumulative excess cases | |
| --- | --- | --- | --- | --- | --- | --- | --- | --- | --- | --- |
|  | Cases (n) | Cumulative cases (n) | Cumulative cases per 1,000 patients [%,lci,uci] | Cases (n) | Cumulative cases (n) | Cumulative cases per 1,000 patients [%,lci,uci] | n | P-value | n | Cases per 1,000 total patients |
| 1 | 856 | 856 | 3.4 [3.2,3.7] | 194 | 194 | 0.8 [0.8,0.8] | 662 | <0.001 | 662 | 2.6 |
| 2 | 649 | 1,505 | 6 [5.7,6.3] | 194 | 388 | 1.5 [1.5,1.6] | 455 | <0.001 | 1117 | 4.5 |
| 3 | 411 | 1,916 | 7.6 [7.3,8] | 194 | 582 | 2.3 [2.3,2.3] | 217 | <0.001 | 1334 | 5.3 |
| 4 | 331 | 2,247 | 9 [8.6,9.3] | 194 | 776 | 3.1 [3.1,3.1] | 137 | <0.001 | 1471 | 5.9 |
| 5 | 293 | 2,540 | 10.1 [9.7,10.5] | 194 | 970 | 3.9 [3.8,3.9] | 99 | <0.001 | 1570 | 6.3 |
| 6 | 276 | 2,816 | 11.2 [10.8,11.7] | 194 | 1,164 | 4.6 [4.6,4.7] | 82 | <0.001 | 1652 | 6.6 |
| 7 | 209 | 3,025 | 12.1 [11.6,12.5] | 194 | 1,358 | 5.4 [5.4,5.4] | 15 | 0.285 | 1667 | 6.7 |
| 8 | 250 | 3,275 | 13.1 [12.6,13.5] | 194 | 1,552 | 6.2 [6.2,6.2] | 56 | <0.001 | 1723 | 6.9 |
| 9 | 222 | 3,497 | 14 [13.5,14.4] | 194 | 1,746 | 7 [6.9,7] | 28 | 0.049 | 1751 | 7.0 |
| 10 | 198 | 3,695 | 14.7 [14.3,15.2] | 194 | 1,940 | 7.7 [7.7,7.8] | 4 | 0.767 | 1755 | 7.0 |
| 11 | 196 | 3,891 | 15.5 [15,16] | 194 | 2,134 | 8.5 [8.5,8.6] | 2 | 0.877 | 1757 | 7.0 |
| 12 | 196 | 4,087 | 16.3 [15.8,16.8] | 194 | 2,328 | 9.3 [9.3,9.3] | 2 | 0.877 | 1759 | 7.0 |
| 13 | 181 | 4,268 | 17 [16.5,17.5] | 194 | 2,522 | 10.1 [10,10.1] | -13 | 0.353 | 1746 | 7.0 |
| 14 | 191 | 4,459 | 17.8 [17.3,18.3] | 194 | 2,716 | 10.8 [10.8,10.9] | -3 | 0.839 | 1743 | 7.0 |
| 15 | 201 | 4,660 | 18.6 [18.1,19.1] | 194 | 2,910 | 11.6 [11.6,11.7] | 7 | 0.611 | 1750 | 7.0 |
| 16 | 216 | 4,876 | 19.5 [18.9,20] | 194 | 3,104 | 12.4 [12.3,12.4] | 22 | 0.120 | 1772 | 7.1 |
| 17 | 194 | 5,070 | 20.2 [19.7,20.8] | 194 | 3,298 | 13.2 [13.1,13.2] | 0 | 1.009 | 1772 | 7.1 |
| 18 | 184 | 5,254 | 21 [20.4,21.5] | 194 | 3,492 | 13.9 [13.9,14] | -10 | 0.478 | 1762 | 7.0 |
| 19 | 184 | 5,438 | 21.7 [21.1,22.3] | 194 | 3,686 | 14.7 [14.7,14.8] | -10 | 0.478 | 1752 | 7.0 |
| 20 | 178 | 5,616 | 22.4 [21.8,23] | 194 | 3,880 | 15.5 [15.4,15.5] | -16 | 0.251 | 1736 | 6.9 |
| 21 | 180 | 5,796 | 23.1 [22.5,23.7] | 194 | 4,074 | 16.3 [16.2,16.3] | -14 | 0.317 | 1722 | 6.9 |
| 22 | 158 | 5,954 | 23.8 [23.2,24.4] | 194 | 4,268 | 17 [17,17.1] | -36 | 0.008 | 1686 | 6.7 |
| 23 | 177 | 6,131 | 24.5 [23.9,25.1] | 194 | 4,462 | 17.8 [17.8,17.9] | -17 | 0.222 | 1669 | 6.7 |
| 24 | 197 | 6,328 | 25.3 [24.6,25.9] | 194 | 4,656 | 18.6 [18.5,18.6] | 3 | 0.822 | 1672 | 6.7 |
| Total patients | 250,606 | | | 250,606 | | |  | |  |  |
| ^a^Cancer diagnoses between 2007-2015, up to 24 months after first presentation with fatigue to primary care in 2007-2013. ^b^Expected cases for the age/ sex distribution of patients with fatigue, based on five-year age band and sex-specific estimated monthly population incidence, using annual number of cancer diagnoses and mid-year population estimates for England, 2011. | | | | | | | | | | |

**Appendix 3. International Classification of Diseases (ICD)-10 codes used to define all cancers combined, and each cancer site**

| Cancer site | ICD10 codes |
| --- | --- |
| All malignant cancers excl. non melanoma skin cancer (including selected non-malignant cancers as specified) | C00-C97 (excluding C44) |
| Bladder | C67 |
| Malignant brain and other CNS | C70-72 |
| Breast | C50 |
| Cancer of unknown primary | C77-80 |
| Cervix | C53 |
| Colorectal | C18, C19, C20 |
| Head & neck | C00-C14, C31, C32 |
| Lymphoma (non-hodgkins & hodgkins) | C81, C82-85 |
| Kidney | C64 |
| Leukaemia | C91-95 |
| Liver | C22 |
| Lung & mesothelioma | C33, C34, C45 |
| Melanoma | C43 |
| Multiple myeloma | C90 |
| Ovary | C56-57 |
| Pancreas | C25 |
| Prostate | C61 |
| Sarcoma (soft tissue, connective & bone) | C40-41, C48-49 |
| Testis | C62 |
| Thyroid | C73 |
| Upper gastro-intestinal | C15-C16 |
| Uterus | C54-55 |
| Vulva | C51 |

**Appendix 4. STROBE Statement—Checklist of items that should be included in reports of *cohort studies***

|  | **Item No** | **Recommendation** | **Section & paragraph number** |  |
| --- | --- | --- | --- | --- |
| **Title and abstract** | 1 | (*a*) Indicate the study’s design with a commonly used term in the title or the abstract | Title/ abstract |  |
|  |  | (*b*) Provide in the abstract an informative and balanced summary of what was done and what was found | Abstract |  |
| **Introduction** | | | |  |
| Background/ rationale | 2 | Explain the scientific background and rationale for the investigation being reported | Background para. 1-3 |  |
| Objectives | 3 | State specific objectives, including any prespecified hypotheses | Background para. 4 |  |
| **Methods** | | | |  |
| Study design | 4 | Present key elements of study design early in the paper | Methods: Study design and data source |  |
| Setting | 5 | Describe the setting, locations, and relevant dates, including periods of recruitment, exposure, follow-up, and data collection | Methods: Study design and data source |  |
| Participants | 6 | (*a*) Give the eligibility criteria, and the sources and methods of selection of participants. Describe methods of follow-up | Methods: Cohort identification |  |
|  |  | (*b*) For matched studies, give matching criteria and number of exposed and unexposed | N/a – no matching |  |
| Variables | 7 | Clearly define all outcomes, exposures, predictors, potential confounders, and effect modifiers. Give diagnostic criteria, if applicable | Methods: Follow up and outcomes; Methods: Statistical analysis para. 1 |  |
| Data sources/ measurement | 8* | For each variable of interest, give sources of data and details of methods of assessment (measurement). Describe comparability of assessment methods if there is more than one group | Methods: Study design and data source |  |
| Bias | 9 | Describe any efforts to address potential sources of bias | Methods: Statistical analysis, para. 1-3 |  |
| Study size | 10 | Explain how the study size was arrived at | Methods: Statistical analysis para. 1 |  |
| Quantitative variables | 11 | Explain how quantitative variables were handled in the analyses. If applicable, describe which groupings were chosen and why | Methods: Follow up and outcomes para. 2; Methods: Statistical analysis para. 1 |  |
| Statistical methods | 12 | (*a*) Describe all statistical methods, including those used to control for confounding | Methods: Statistical analysis |  |
|  |  | (*b*) Describe any methods used to examine subgroups and interactions | Methods: Statistical analysis para. 1 |  |
|  |  | (*c*) Explain how missing data were addressed | N/a – no missing |  |
|  |  | (*d*) If applicable, explain how loss to follow-up was addressed | Methods: Follow up and outcomes para. 1 |  |
|  |  | (*e*) Describe any sensitivity analyses | Methods: Cohort identification para. 3-4 |  |
| **Results** | | |  |  |
| Participants | 13* | (a) Report numbers of individuals at each stage of study—eg numbers potentially eligible, examined for eligibility, confirmed eligible, included in the study, completing follow-up, and analysed | Results: Cohort description |  |
|  |  | (b) Give reasons for non-participation at each stage | Results: Cohort description |  |
|  |  | (c) Consider use of a flow diagram | Results: Cohort description Fig. 1 |  |
| Descriptive data | 14* | (a) Give characteristics of study participants (eg demographic, clinical, social) and information on exposures and potential confounders | Results: Cohort description & Table 1. |  |
|  |  | (b) Indicate number of participants with missing data for each variable of interest | N/a – no missing |  |
|  |  | (c) Summarise follow-up time (eg, average and total amount) | N/a – complete follow up |  |
| Outcome data | 15* | Report numbers of outcome events or summary measures over time | Results: Risk of cancer & Table 2. |  |
| Main results | 16 | (*a*) Give unadjusted estimates and, if applicable, confounder-adjusted estimates and their precision (eg, 95% confidence interval). Make clear which confounders were adjusted for and why they were included | Findings: Risk of cancer; Findings: Frequency of specific cancer sites; Findings: Distribution of incident cases by month following recorded fatigue | |
|  |  | (*b*) Report category boundaries when continuous variables were categorized | N/a – no continuous variables | |
|  |  | (*c*) If relevant, consider translating estimates of relative risk into absolute risk for a meaningful time period | Findings: Table 2, Table 3, Fig. 2, Fig. 3 | |
| Other analyses | 17 | Report other analyses done—eg analyses of subgroups and interactions, and sensitivity analyses | Findings: Sensitivity analyses | |
| **Discussion** | | | | |
| Key results | 18 | Summarise key results with reference to study objectives | Discussion: Key findings | |
| Limitations | 19 | Discuss limitations of the study, taking into account sources of potential bias or imprecision. Discuss both direction and magnitude of any potential bias | Discussion: Strengths and limitations | |
| Interpretation | 20 | Give a cautious overall interpretation of results considering objectives, limitations, multiplicity of analyses, results from similar studies, and other relevant evidence | Discussion: Implications | |
| Generalisability | 21 | Discuss the generalisability (external validity) of the study results | Discussion: Strengths and limitations para. 3 | |
| **Other information** | | | | |
| Funding | 22 | Give the source of funding and the role of the funders for the present study and, if applicable, for the original study on which the present article is based | Additional information: Funding information | |

*Give information separately for exposed and unexposed groups.

**Note:** An Explanation and Elaboration article discusses each checklist item and gives methodological background and published examples of transparent reporting. The STROBE checklist is best used in conjunction with this article (freely available on the Web sites of PLoS Medicine at http://www.plosmedicine.org/, Annals of Internal Medicine at http://www.annals.org/, and Epidemiology at http://www.epidem.com/). Information on the STROBE Initiative is available at http://www.strobe-statement.org.

**Appendix 5a. Deprivation quintile of patients presenting to primary care with fatigue, compared to England, by gender**

|  | Men | | | | | Women | | | | |
| --- | --- | --- | --- | --- | --- | --- | --- | --- | --- | --- |
|  | Patients with fatigue | | England population^b^ | | | Patients with fatigue | | | England population^b^ | |
|  | N | % | N | | % | N | % | | N | % |
| Deprivation quintile^a^ |  |  |  | |  |  |  | |  |  |
| 1 - least deprived | 18,961 | 23.37 | 2,861,959 | | 17.90 | 39,462 | 23.29 | | 3,039,573 | 17.72 |
| 2 | 18,639 | 22.97 | 3,117,552 | | 19.50 | 37,675 | 22.23 | | 3,299,259 | 19.23 |
| 3 | 17,604 | 21.70 | 3,301,569 | | 20.65 | 36,178 | 21.35 | | 3,539,247 | 20.63 |
| 4 | 14,084 | 17.36 | 3,355,807 | | 20.99 | 30,086 | 17.75 | | 3,627,502 | 21.14 |
| 5 - most deprived | 11,793 | 14.53 | 3,349,259 | | 20.95 | 25,966 | 15.32 | | 3,651,392 | 21.28 |
| Missing | 62 | 0.08 | - | | - | 96 | 0.06 | | - | - |
| Total people | 81,143 |  | 15,986,146 |  | | 169,463 | |  | 17,156,973 |  |
| ^a^Index of Multiple Deprivation (IMD) quintile of the person's area of residence. ^b^Published statistics for men/ women aged 30 years and over in England in 2011, available at: [www.ons.gov.uk/peoplepopulationandcommunity/populationandmigration/populationestimates/adhocs/12386populationbyindexofmultipledeprivationimdengland2001to2019](http://www.ons.gov.uk/peoplepopulationandcommunity/populationandmigration/populationestimates/adhocs/12386populationbyindexofmultipledeprivationimdengland2001to2019) | | | | | | | | | | |

**Appendix 5b. Number and proportion of patients diagnosed with cancer within a year after presenting to primary care with fatigue, by gender and index of multiple deprivation**

|  | Men | | | Women | | |
| --- | --- | --- | --- | --- | --- | --- |
|  | Cancer^a^ | | Total | Cancer^a^ | | Total |
|  | n | % [lci,uci] | N | n | % [lci,uci] | N |
| Deprivation quintile^b^ |  |  |  |  |  |  |
| 1 - least deprived | 474 | 2.5 [2.28,2.74] | 18,961 | 465 | 1.18 [1.07,1.29] | 39,462 |
| 2 | 458 | 2.46 [2.24,2.69] | 18,639 | 491 | 1.3 [1.19,1.42] | 37,675 |
| 3 | 416 | 2.36 [2.14,2.6] | 17,604 | 465 | 1.29 [1.17,1.41] | 36,178 |
| 4 | 334 | 2.37 [2.12,2.64] | 14,084 | 370 | 1.23 [1.11,1.36] | 30,086 |
| 5 - most deprived | 302 | 2.56 [2.28,2.87] | 11,793 | 308 | 1.19 [1.06,1.33] | 25,966 |
| ^a^Cancer diagnoses between 2007-2014, 12 months after first presentation, for patients presenting to primary care with a valid fatigue symptom between 2007-2013. ^b^Index of Multiple Deprivation (IMD) quintile of the person's area of residence. Data not shown for missing IMD to reduce statistical disclosure risk. | | | | | | |

**Appendix 6a. Records of specific fatigue read codes, including all chronic fatigue syndrome and post-viral fatigue syndrome codes, as a proportion of all eligible records of fatigue between 2007-2013**

| Read code | Read code description | Number of records | Proportion of total records of fatigue |  |
| --- | --- | --- | --- | --- |
|  |  | n | % | |
| 168..00 | Tiredness symptom | 100,126 | 25.97 | |
| 1683.00 | Tired all the time | 85,050 | 22.06 | |
| R007500 | [D]Tiredness | 29,903 | 7.76 | |
| 1683.11 | C/O - 'tired all the time' | 23,822 | 6.18 | |
| 168..12 | Lethargy - symptom | 23,136 | 6.00 | |
| 1B3..12 | Weakness symptoms | 20,220 | 5.24 | |
| 1682.00 | Fatigue | 19,410 | 5.03 | |
| E205.12 | Tired all the time | 18,298 | 4.75 | |
| 168..11 | Fatigue - symptom | 17,016 | 4.41 | |
| R007000 | [D]Malaise | 12,429 | 3.22 | |
| R007300 | [D]Lethargy | 10,182 | 2.64 | |
| *F286.11 | CFS - Chronic fatigue syndrome | 3,024 | 0.78 | |
| R007100 | [D]Fatigue | 2,053 | 0.53 | |
| *F286.00 | Chronic fatigue syndrome | 1,871 | 0.49 | |
| 1684.00 | Malaise/lethargy | 1,642 | 0.43 | |
| R2y3.00 | [D]Debility, unspecified | 1,527 | 0.40 | |
| 1688.00 | Exhaustion | 1,489 | 0.39 | |
| 1B32.00 | Weakness present | 1,265 | 0.33 | |
| R007z11 | [D]Lassitude | 1,235 | 0.32 | |
| 1684.11 | C/O - debility - malaise | 1,054 | 0.27 | |
| 168Z.00 | Tiredness symptom NOS | 692 | 0.18 | |
| **F286.12 | Postviral fatigue syndrome | 636 | 0.16 | |
| R007211 | [D]General weakness | 567 | 0.15 | |
| E205.11 | Nervous exhaustion | 548 | 0.14 | |
| R007200 | [D]Asthenia NOS | 407 | 0.11 | |
| **R007400 | [D]Postviral (asthenic) syndrome | 353 | 0.09 | |
| E205.00 | Neurasthenia - nervous debility | 295 | 0.08 | |
| 2832.12 | O/E - weakness | 271 | 0.07 | |
| **F286.14 | Post-viral fatigue syndrome | 220 | 0.06 | |
| Eu46011 | [X]Fatigue syndrome | 169 | 0.04 | |
| R007z00 | [D]Malaise and fatigue NOS | 113 | 0.03 | |
| 2254.00 | O/E - apathetic | 94 | 0.02 | |
| **R007411 | [D]Post viral debility | 89 | 0.02 | |
| **F286.13 | PVFS - Postviral fatigue syn | 62 | 0.02 | |
| Eu46000 | [X]Neurasthenia | 19 | 0.00 | |
| R204.00 | [D]Senile exhaustion | 12 | 0.00 | |
| R007.00 | [D]Malaise and fatigue | 10 | 0.00 | |
| *F286100 | Moderate chronic fatigue syndrome | <5^a^ | - | |
| *F286200 | Severe chronic fatigue syndrome | <5^a^ | - | |
| *F286000 | Mild chronic fatigue syndrome | <5^a^ | - | |
| *All chronic fatigue syndrome codes | | 4,895 | 1.27 | |
| **All post-viral fatigue syndrome codes | | 1,360 | 0.35 | |
| All records of fatigue | | 385,564 | | |
| ^a^Cell counts under 5 are suppressed to reduce statistical disclosure risk. | | | | |

**Appendix 6b. Number and proportion of patients whose index fatigue presentation was CFS or PVFS, by gender**

|  | Men | | Women | |
| --- | --- | --- | --- | --- |
|  | n | % | n | % |
| Chronic fatigue syndrome (CFS) only | 356 | 0.44 | 903 | 0.53 |
| Post-viral fatigue syndrome (PVFS) only | 240 | 0.30 | 534 | 0.32 |
| CFS or PVFS | 596 | 0.73 | 1,437 | 0.85 |
| All patients with fatigue including CFS & PVFS | 81,143 | | 169,463 | |
| ^a^Patients presenting to primary care with a valid fatigue symptom between 2007-2013. | | | | |

**Appendix 6c. Number and proportion of patients diagnosed with cancer within a year after presenting to primary care with fatigue, excluding patients whose index fatigue presentation was CFS or PVFS, by gender**

|  | Cancer^a^ | | Total patients |
| --- | --- | --- | --- |
|  | n | % | N |
| Chronic fatigue syndrome (CFS) only | 6 | 0.48 | 1,259 |
| Post-viral fatigue syndrome (PVFS) only | 5 | 0.65 | 774 |
| All fatigue codes excluding CFS & PVFS | 4,076 | 1.64 | 248,573 |
| All fatigue codes including CFS & PVFS | 4,087 | 1.63 | 250,606 |
| ^a^Cancer diagnoses between 2007-2014, 12 months after first presentation, for patients presenting to primary care with a valid fatigue symptom between 2007-2013. Results shown for men and women combined to reduce statistical disclosure risk. | | | |

**Appendix 7. Risk of subsequent cancer diagnosis within 3-24 months after first (index) presentation to primary care with fatigue, including versus excluding eligible fatigue presentations with a previous ‘ineligible’ fatigue presentation or cancer diagnosis in the previous one or two years.**

| Subsequent cancer | Look-back period: one year^a^ | | | | | | | | Look-back period: two years^b^ | | | | | | | |
| --- | --- | --- | --- | --- | --- | --- | --- | --- | --- | --- | --- | --- | --- | --- | --- | --- |
|  | Including patients with a previous ‘ineligible’ fatigue presentation or cancer diagnosis | | Excluding previous ‘ineligible’ fatigue presentation | | Excluding previous cancer diagnosis | | Excluding previous ‘ineligible’ fatigue presentation or cancer diagnosis | | Including patients with a previous ‘ineligible’ fatigue presentation or cancer diagnosis | | Excluding previous ‘ineligible’ fatigue presentation | | Excluding previous cancer diagnosis | | Excluding previous ‘ineligible’ fatigue presentation or cancer diagnosis | |
|  | n | % | n | % | n | % | n | % | n | % | n | % | n | % | n | % |
| Within 3 months | 2,004 | 0.8% | 1,981 | 0.8% | 1,941 | 0.8% | 1,916 | 0.8% | 1,705 | 0.8% | 1,658 | 0.8% | 1,634 | 0.7% | 1,585 | 0.8% |
| Within 6 months | 2,921 | 1.1% | 2,892 | 1.1% | 2,848 | 1.1% | 2,816 | 1.1% | 2,514 | 1.1% | 2,434 | 1.1% | 2,422 | 1.1% | 2,343 | 1.1% |
| Within 12 months | 4,233 | 1.7% | 4,182 | 1.7% | 4,142 | 1.6% | 4,087 | 1.6% | 3,652 | 1.6% | 3,526 | 1.7% | 3,530 | 1.6% | 3,400 | 1.6% |
| Within 18 months | 5,431 | 2.1% | 5,366 | 2.1% | 5,325 | 2.1% | 5,254 | 2.1% | 4,715 | 2.1% | 4,541 | 2.1% | 4,569 | 2.1% | 4,392 | 2.1% |
| Within 24 months | 6,520 | 2.5% | 6,452 | 2.5% | 6,404 | 2.5% | 6,328 | 2.5% | 5,689 | 2.5% | 5,467 | 2.6% | 5,529 | 2.5% | 5,301 | 2.5% |
| Total patients | 256,865 |  | 253,592 |  | 254,026 |  | 250,606 |  | 224,254 |  | 213,865 |  | 219,947 |  | 209,406 |  |
| ^a^Cancer diagnoses between 2007-2015, for patients presenting to primary care with fatigue in 2007-2013. ^b^Cancer diagnoses between 2008-2015, for patients presenting to primary care with fatigue in 2008-2013. | | | | | | | | | | | | | | | | |
